# Coping with Viral Diversity in HIV Vaccine Design

**DOI:** 10.1371/journal.pcbi.0030075

**Published:** 2007-04-27

**Authors:** David C Nickle, Morgane Rolland, Mark A Jensen, Sergei L. Kosakovsky Pond, Wenjie Deng, Mark Seligman, David Heckerman, James I Mullins, Nebojsa Jojic

**Affiliations:** 1 Department of Microbiology, University of Washington School of Medicine, Seattle, Washington, United States of America; 2 Department of Pathology, University of California San Diego, La Jolla, California, United States of America; 3 Microsoft Research, Redmond, Washington, United States of America; ETH Zürich, Switzerland

## Abstract

The ability of human immunodeficiency virus type 1 (HIV-1) to develop high levels of genetic diversity, and thereby acquire mutations to escape immune pressures, contributes to the difficulties in producing a vaccine. Possibly no single HIV-1 sequence can induce sufficiently broad immunity to protect against a wide variety of infectious strains, or block mutational escape pathways available to the virus after infection. The authors describe the generation of HIV-1 immunogens that minimizes the phylogenetic distance of viral strains throughout the known viral population (the center of tree [COT]) and then extend the COT immunogen by addition of a composite sequence that includes high-frequency variable sites preserved in their native contexts. The resulting COT^+^ antigens compress the variation found in many independent HIV-1 isolates into lengths suitable for vaccine immunogens. It is possible to capture 62% of the variation found in the Nef protein and 82% of the variation in the Gag protein into immunogens of three gene lengths. The authors put forward immunogen designs that maximize representation of the diverse antigenic features present in a spectrum of HIV-1 strains. These immunogens should elicit immune responses against high-frequency viral strains as well as against most mutant forms of the virus.

## Author Summary

The ability of human immunodeficiency virus type 1 (HIV-1) to acquire mutations that preserve virus viability yet evade immune responses contributes to the current failure in producing a vaccine. We describe the generation of candidate HIV-1 immunogens that include multiple forms of variable elements of the virus including some that retain colinearity with the virus and thus are expected to retain protein function. These antigens compress the variation found in many viral strains into lengths suitable for vaccine immunogens. For example, we can capture 62% of the variation found in the Nef protein and 82% of the variation in the Gag protein into immunogens of three gene lengths. We put forward immunogen designs that maximize representation of the diverse antigenic features present in a spectrum of HIV-1 strains. These immunogens should elicit immune responses against high frequency viral strains as well as against most mutant forms of the virus.

## Introduction

The failure of AIDS vaccine efforts in the past 20-plus years is thought to be due, in part, to the enormous viral antigenic diversity found within and among patients with human immunodeficiency virus type 1 (HIV-1) infection. However, until recently, relatively little effort had been devoted to choosing particular viral variant sequences or designing sequences to include within vaccines [1,2]. There were early attempts to design vaccines by concatenating commonly recognized T cell and antibody epitopes [3], but these did not produce a viable vaccine candidate. New methods of combining epitopes are being explored in vaccine design, including production of pseudoprotein strings of T cell epitopes [4], and the synthetic scrambled antigen vaccine (SAVINE) [5], which employs consensus overlapping peptide sets from HIV-1 proteins scrambled together. Focusing on the use of whole viral protein sequences, natural strains (NSs) as well as consensus (CON) sequences are being used as a means to minimize the abrogating effect of antigenic diversity in vaccine antigens [2,6,7], as are the inferred most recent common ancestors (MRCA, or ANC) [6,8–10] of targeted virus populations defined as sequences that reside at the basal node of the set of in-group sequences in a phylogenetic tree reconstruction [11]. HIV-1 *env* sequences representing both the CON and ANC have been prepared and studied, but neither has generated exceptionally broad humoral immune reactivity in initial small animal studies [7,12].

In an effort to develop antigens that capture both the summary of circulating variation found in CON estimates, and the coupling of mutations generated with inferred ANC sequences, we have developed an alternative computational method that reconstructs the ancestral state sequence at the center of tree (COT) ([13] and Rolland M, Jensen MA, Nickle DC, Learn GH, Heath L, et al., unpublished data). The COT sequence explicitly minimizes genetic distance, as does the CON, and because it is derived from a phylogenetic tree, it embodies the most likely mutational coupling relationships found in the ANC. Despite these efforts, it may be that no single unit-length antigen, including any NS, CON, ANC, or COT, will encompass sufficient antigenicity to elicit protective immune responses against a broad array of viruses [7,12], as will be required of an AIDS vaccine. This led us to hypothesize that we would need more than one antigenic sequence, or greater than one gene length of the antigen, to elicit protection against the broad antigenic diversity encountered in natural infections. However, cocktails of large numbers of native, full-length NS antigens would quickly become unmanageably complex for practical use as vaccines.

Here, we propose a means to cope with HIV-1 diversity by engineering vaccine antigen constructs to include short protein sequences present at high frequencies in natural viral populations. Currently, this method is explicitly directed toward developing CD8^+^ cytotoxic T lymphocyte (CTL) responses, which are critical to controlling viremia during infection [14–17]. Because the cumulative strength of the CTL-mediated immune response depends on the presence of recognizable epitopes (often approximately nine amino acids in length) in the target proteins, it is logical to seek to maximize epitope coverage within a vaccine antigen. However, although substantial, our current catalog of known CTL epitopes appears to be woefully incomplete [18], hence our strategy relies on the universe of HIV sequences and not solely on known epitope content. Thus, here we will define *coverage* as the sum of the frequencies of all nine amino acid segments (9mers) where the frequency is derived from random independent HIV-1 subtype B isolates found in the vaccine construct. As our epitope catalog increases and our knowledge of protein degradation, CTL epitope binding, and HLA presentation is expanded, this epitope-specific data can be integrated into the measure of coverage (e.g., by weighting epitope frequencies in accordance to their relative “importance” when computing coverage). In this study, we applied our method to Nef because it is highly variable and is potentially very difficult to design an immunogen against, and to Gag because it is immunologically important yet more conserved. We considered subtype B sequences because more immunological information is available about this subtype than any other. This clearly makes the vaccine construct described here as region-specific because of the biogeographic nature of the distribution of viral subtypes across the globe [19]. However, our purpose is to illustrate and demonstrate that this method has promise at producing a vaccine against highly variable infectious agents such as HIV.

## Methods

Vaccination with all known viral sequences would capture all known viral sequence variation, but realistic vaccine constructs might at best include several sequence lengths, each length containing major variants for immune presentation. To quantify variant representation and rationally choose the included variation on this basis, Jojic and colleagues have proposed a method based on machine-learning for the compression of sequence variation into a sequence of minimal length (the “epitome”; [20,21]). Below, we describe an alternative, more transparent algorithm also designed to attain optimized sequence coverage over a fixed-length antigen. We refer to the constructs generated by our method as COT^+^ because they consist of COT antigens augmented by the addition of high-frequency 9mers. We demonstrate the performance of our approach on the highly variable and epitope-rich viral Nef protein as well the epitope-rich major structural protein, Gag. The algorithm consists of five steps applied to a sample of viral nucleotide sequences, each isolated from a separate patient. We started with all publicly available *nef* and *gag* gene sequences from HIV-1 subtype B [22]. By excluding sequences with more than two stop codons and with large indels, and including only independent single sequences from a given individual to avoid sampling bias, we obtained a 169-sequence dataset for Gag; the Nef data set was also constrained to 169 sequences for comparative purposes (Table 1 includes the GenBank IDs of all sequences used). The algorithm, however, can rapidly process datasets with thousands of sequences when such datasets become available.

**Table 1:**
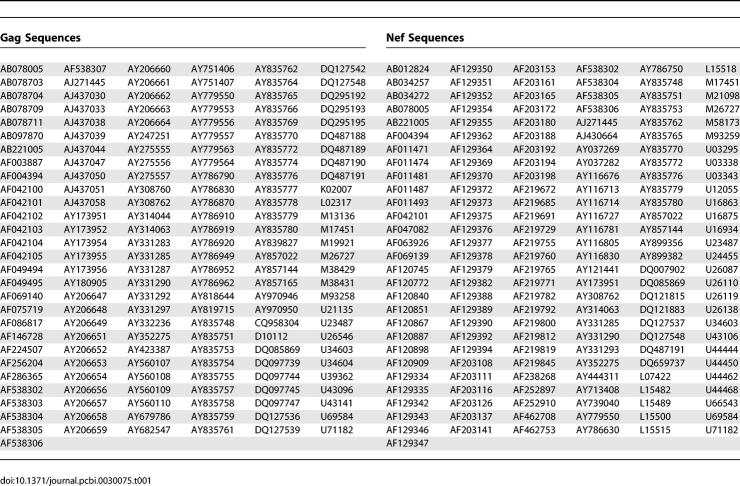
GenBank IDs of Sequences Used

### The Algorithm

**Figure 1:**
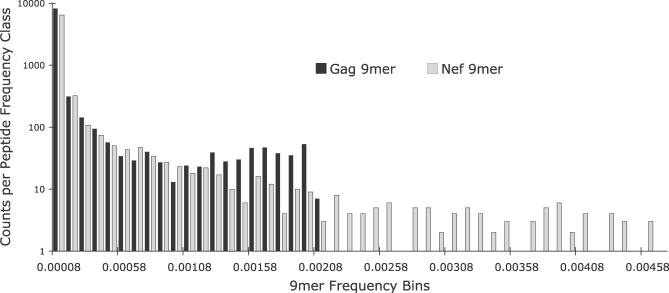
9mer Peptide Distribution Derived from 169 HIV-1 Subtype B Gag and Nef Protein Sequences Each bin in the histogram represents the number of 9mers from a particular frequency class plotted on a log scale. There are only a few peptides found at high frequencies, whereas most of the 9mers occur only once or twice. The score of a given frame is the sum of the frequencies of each unique 9mer contained by the frame. The possible extreme value frequencies for each peptide from all rare to all common is 1.198 × 10^−5^ − 0.0020 for Gag (black bars) and 2.988 × 10^−5^ − 0.0051 for Nef (gray bars). The differences in the two distributions can be explained by the differences in gene length and levels of conservation.

**Figure 2:**
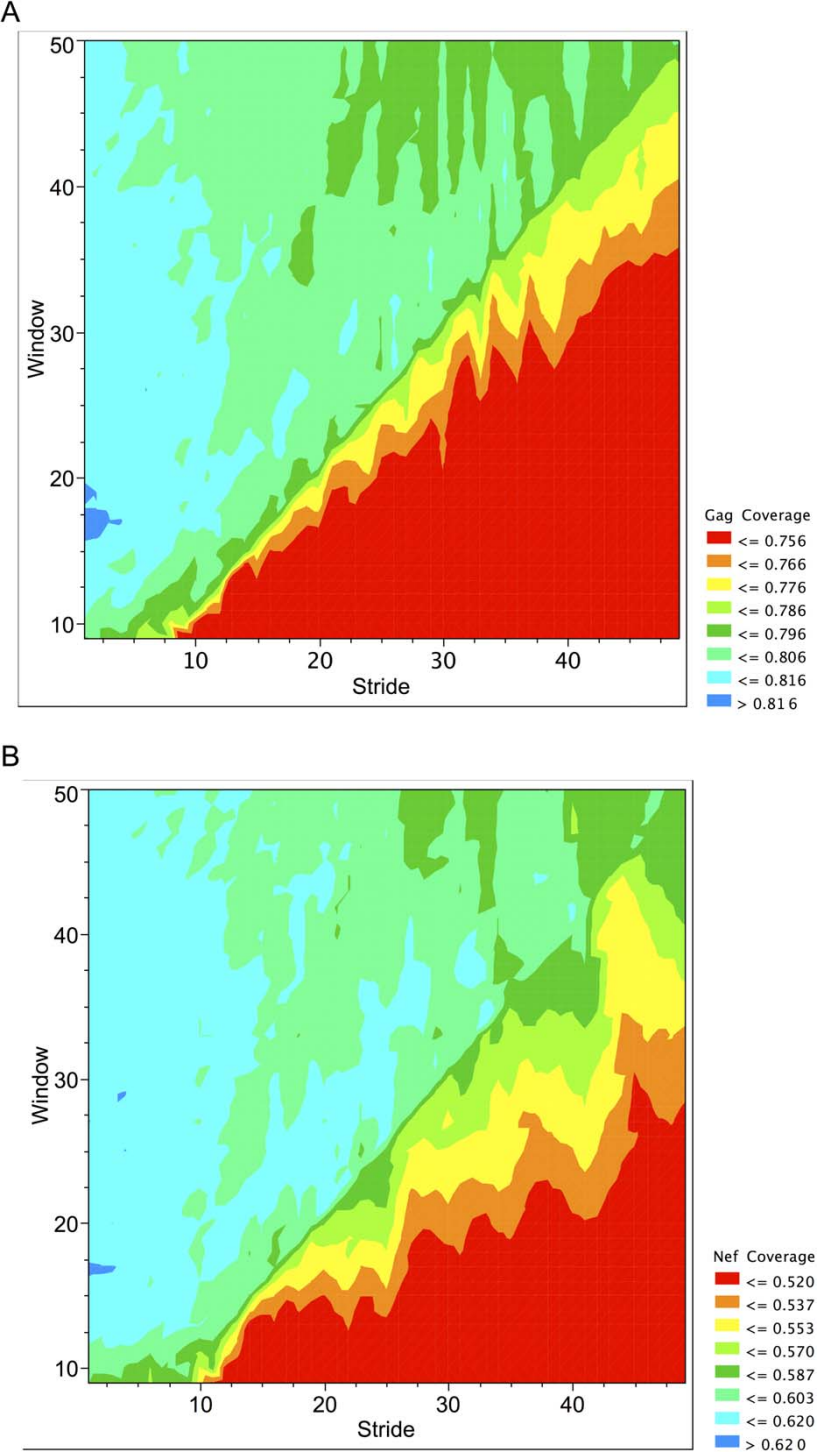
The Effects of Stride versus Window Length on the Measure of Coverage In each graph a three-gene-length COT^+^ construct is evaluated for coverage. Cold (blue) colors indicate high levels of coverage, and hot (red) colors indicate low levels of coverage. The diagonal in the topography represents the transition from strides shorter than window length to strides longer than window length. The maximal coverage at three gene lengths occurs with a window size of 17 with a stride of 1 with no smoothing for both genes—where 82% of the 9mer area is captured for Gag (A) and 62% of the 9mer area is captured for Nef (B). It should be noted that in the area of window of 17 and a stride of 1 the surface is quite flat, and there are several pairs of parameters that give similar results.

(1) A COT sequence is calculated as described ([13 and Rolland M, Jensen MA, Nickle DC, Learn GH, Heath L, et al., unpublished data) from a phylogenetic tree that captures the relationships among genes in the sample using maximum likelihood methods [23]. Briefly, from aligned sequences we estimate a maximum likelihood tree under a HKY + Γ + I model of evolution in PAUP*v4beta10 [24]. The resulting tree is re-rooted at the point that describes the least-squares distance to all the tips on the phylogeny (the COT node). We then infer the maximum likelihood state using the same model of evolution as above.

(2) A table of unique 9mer peptides [20,21] with their corresponding frequencies (the 9mer distribution) is constructed from translated protein sequences. To illustrate this, note that if our sample contained *N* identical sequences of length *L* each, but every 9mer in the COT peptide library was unique, then each peptide would be at equal frequency 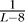 . On the other hand, if every sequence were different from all others, to the extent that no 9mer was represented twice, the frequency for each peptide would equal 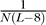 . Actual samples will yield an intermediate distribution that can be exploited for vaccine design (see Figure 1). We used this distribution to compute “coverage”; that is, as we select candidate fragments to be included in the potential vaccine, we will select only those fragments that are highly represented under the 9mer curve.

(3) Unique or rare 9mers, which by definition are unlikely to be common in circulating viral strains, are likely to derive from low-fitness variants [25,26] and, because of their low frequency, have low probability of being incorporated in our vaccine constructs. Specifically, we calculate the frequency of all observed mutations at each site, and revert any mutation with a frequency below a fixed “smoothing” threshold, *M*, to the corresponding character in the COT sequence. All 9mers present in the COT sequence are then removed from the 9mer distributions before proceeding to the next step.

(4) Given a fixed window size *F* (ranging from 9 to *L*, where *L* = the length of the protein sequence [we start with 9 because that is the size of the peptide that is most often found to encode epitope sequences] and a stride parameter *S* [ranging from 1 to *L*, the length of the protein]), we generate all sequence fragments from the sampled sequence by iteratively shifting the frame *S* residues at a time. We then compute the coverage for each sequence fragment not already present in the COT sequence, and append the sequence fragment to the COT string, compressing with possible overlap to yield a COT^+^ molecule with the highest ratio of coverage per length. Specifically, fragments are chosen by their level of coverage and whether or not they have differences with respect to the COT sequences. The highest coverage fragments are chosen first, with subsequent fragments with lower coverage being chosen subsequently. This process is repeated until the sequence of desired length is derived. The length of the COT^+^ sequence is arbitrarily chosen by taking into account plasmid size limitations for producing and delivering an antigen construct and the amount of variability that can be efficiently incorporated as the length is extended, which in turn depends on the variability found in circulating strains that have been sampled for a particular gene. We note that it is possible to arrange the order in which sequence fragments are added to COT^+^ to maximize the overlap of consecutive fragments, thereby further compressing the antigen.

(5) The values of window size *F,* stride step *S,* and smoothing threshold *M* are varied to achieve maximum coverage (Figure 2A and 2B).

### Comparison with Random Sequences

**Figure 3:**
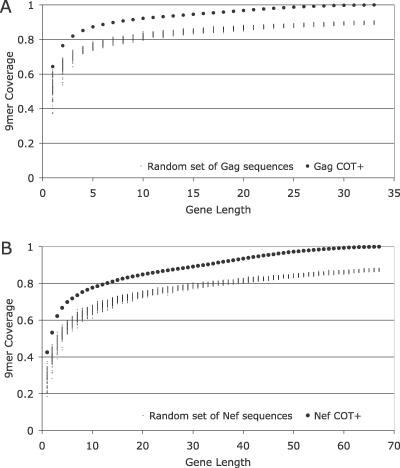
Coverage Comparison between COT^+^ and 100 Randomly Sampled (without Replacement) Sets of Sequences of the Same Length The comparison at the single gene length is for HIV-1 subtype B Gag and Nef, and measures the COT sequence against randomly sampled database sequences. The COT^+^ captures all known variation in the training dataset at 33 gene lengths for Gag (A) and 67 gene lengths for Nef (B). Neither Gag nor Nef randomly sampled datasets will reach 100% coverage until 100% of the data is sampled.

We compared our constructs of various lengths to randomly drawn sequences from the curated dataset of 169 sequences using the optimal values for *F* and *S*. We generated COT^+^ for both Gag and Nef at ever-increasing unit protein lengths until we reached 100% coverage. For comparison, we concatenated randomly sampled protein sequences 100 times at ever-increasing unit protein lengths from both Gag and Nef and measured 9mer coverage across the same gene lengths (Figure 3A and 3B). We chose protein unit length for our comparison, but COT^+^ can be derived for any partial unit protein length desired.

### Cross-Validation

To ensure that we were not overestimating the coverage of our constructs due to the finite size of our dataset, we repeated our approach using 10-fold cross-validation. We partitioned the data into ten sets, and for each we estimated COT^+^ from the remaining 90% of the data and then measured its coverage of the sequences in the chosen set. Thus, given that our assessment of coverage is on a set of sequences not seen in training, we yield an estimated lower bound on the coverage we would obtain for a larger population. We report this lower bound as a percentage of similarity to the estimated upper-bound COT^+^, derived from training and testing on all 169 sequences for both Gag and Nef. This study is geared to understand the effect of sample size on the on the COT^+^ estimation and to show that we are not overfitting the estimations.

## Known Epitope Coverage

Although the list of known HIV-specific CD8 T-cell epitopes is far from complete [18], we sought to determine how well our 9mer coverage-based constructs identified known epitopes. To this end, we obtained all available HIV CTL epitopes from the Los Alamos National Laboratory (LANL) HIV immunology database [27] and counted the perfect matches to our constructs. Because many true epitopes are listed multiple times and larger peptides are reported frequently where the true epitope is embedded, we curated the database to remove any larger epitope that had a smaller embedded known epitope with the same supertype HLA response pattern, and removed any duplicates.

## Results

We inferred COT sequences from databases of Gag and Nef protein sequences from HIV-1 subtype B from 169 independently infected individuals, and then added frequently observed variant 9mer peptides to create COT^+^ sequences. The frequencies of unique 9-mer peptides are shown in Figure 1. We find that maximal coverage occurs when the window size, *F,* is 17, the stride length, *S,* is 1, and when smoothing *M* is 0 (Figure 2A and 2B). One possible reason for why an *S* value equal to 1 leads to the highest coverage is that it gives every amino acid in the sequences a chance to be in every possible position in a high-scoring peptide. Counterintuitive to this is the observation that *S* values greater than 1 do not get penalized with big drops in 9mer coverage. We think the explanation for this observation has to do with the fact that even with *S* larger than 1, every amino acid in the sequences is still considered when building a construct. This is exemplified by the fact that the biggest drops in 9mer coverage come when *S* is larger than *F,* because it is in this parameter space that some amino acids have the probability of not being considered at all in the resulting construct.

Adding peptides to generate a three-gene-length COT^+^ construct achieved 82% 9mer coverage for Gag and 62% for Nef, whereas an antigen constructed from several random concatenated database sequences [22] needed to achieve the same level of coverage required ten gene lengths for Gag and approximately 11 for Nef (Figure 3A and 3B). When COT^+^ is compared with 100 constructs of the same length obtained by concatenating randomly selected sequences from the Los Alamos National Laboratory database [22], the COT^+^ estimate had a higher level of coverage in every case (randomization test, *p* < .01) for both Gag and Nef. The flattening of the curves in Figure 3A and 3B suggests that after the COT^+^ construct has grown past a few gene lengths, the benefit of adding more length is dramatically reduced. For example, the extension of the COT^+^ construct from one to three gene lengths results in a 16% increase in coverage for Gag and a 13% increase in coverage for Nef. However, extending COT^+^ from three to five gene lengths yields only 5% additional coverage for both Gag and Nef. The COT^+^ sequence reaches 100% coverage at 33 gene lengths for Gag and 67 gene lengths for Nef, while the randomly sampled sets reach 100% coverage only after all 169 sequences are included. The latter observation is due to the fact that many of the mutations found in HIV are private (i.e., found only within the lineage infecting a particular person).

When applied to small datasets, our algorithm generates COT^+^ constructs with high coverage. An extreme example is making a three-gene construct from just three genes in the training set. In this scenario, we can trivially achieve 100% coverage. The larger the training set, the lower the coverage in a three-gene-length vaccine construct. A 10-fold cross-validation study was therefore designed to determine the effects of sample size on our COT^+^ constructs. Specifically, at three protein lengths, the cross-validated coverage of Gag and Nef are 96% and 93%, respectively. This suggests that for both proteins these inferences are generalizable across HIV-1 subtype B and that adding more sequence data into the training dataset would add very little to these estimations. That is to say, 10% of the original 169 sequences produce estimations of the COT^+^ that are highly consistent with the estimations from the entire dataset, supporting the notion that there is a saturation effect and that adding sequences beyond the 169 will not give rise to better estimations.

Assessing the inclusion of functional CTL epitopes in our constructs is problematic. The majority of the known CTL epitopes were mapped using peptides derived from a limited number of HIV strains (e.g., laboratory-adapted strains and consensus sequences). The CTL database is also incomplete (e.g., a recent study that used a subset of autologous peptides from a single patient enabled recognition of 28% more epitopes in the virus than were previously reported [18]), and it is unclear whether characterized epitopes form an unbiased sample of naturally occurring antigenic peptides. It is also necessary that the epitope be presented in the proper context of adjacent amino acids for efficient immunoproteasome cleavage. We therefore assessed the overall size of the peptides needed to obtain maximal coverage of included 9mers. As shown in Figure 2A and 2B, maximal coverage of both the Gag and Nef datasets was obtained with a window size of 17 amino acids and a stride of one amino acid and no smoothing required (see Methods). Hence, we are able to construct immunogens that preserve much of the extended local amino acid environment of the epitope without sacrificing coverage. This enhances the likelihood that the desired peptide epitope will be properly cleaved by cellular proteases and presented efficiently on HLA molecules.

**Figure 4:**
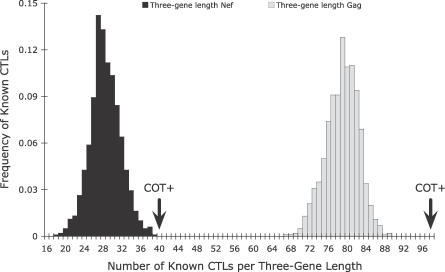
The Distribution of the Number of Known Epitopes in Three Randomly Chosen *Gag* (Right-Side Distribution) and *Nef* (Left-Side Distribution) Genes from the Los Alamos National Laboratory Database The COT^+^ sequence at three gene lengths for Gag has 98 out of 102 known CTL epitopes, and Nef has 40 out of 49 known CTL epitopes.

Next, we assessed the inclusion of known CTL epitopes in our constructs by comparing the number of known HIV-1 Nef and Gag epitopes [27] contained in the three-gene-length COT^+^ constructs to that of 1,000 combinations of three randomly selected database sequences (Figure 4). *S*equences from the viral strains used to map these epitopes were excluded from the randomization study. Although our algorithm does not attempt to explicitly enrich for known CTL epitopes, the number of known epitopes in COT^+^ is significantly higher than in a random three-gene construct (*p* < 0.001) for both Gag and Nef. This suggests that COT^+^ provides a substantial boost in the number of epitopes shared between the immunogen and a random circulating database variant, and thus may have enhanced potential as an immunogen.

## Discussion

COT^+^ constructs provide a means to extensively compress epitope variation into an immunogen of minimal size. Much of the known variation of both the relatively conserved HIV-1 Gag gene and the quite variable Nef gene can be successfully compressed into COT^+^ constructs of a few gene lengths. Little increases in variation coverage are noted, however, beyond three to four gene lengths. Coverage grows with length approximately in a *y* = *m*log(*x*) + *b* form where *y* is coverage and *x* is length of the construct. The difference between COT^+^ construct of Gag and Nef can be broken down into these terms. The coverage intercept parameter *b* is higher for Gag constructs than for Nef simply because Gag is a more conserved protein than Nef. However, the parameter *m* is larger for Nef than it is for Gag because the benefits of 9mer compression on coverage are higher with constructs made from variable proteins.

Our COT^+^ generation algorithm is a rapid, computationally efficient heuristic approximation, though it is not guaranteed to find the antigen that achieves maximal epitope coverage for a fixed length. More computationally intensive approaches, such as genetic algorithm searches or approximate solutions to the classic Traveling Salesman problem (see http://mathworld.wolfram.com/TravelingSalesmanProblem.html), could also be brought to bear on the problem of antigen design. Surprisingly, selecting the high-frequency 9mers alone and appending them to the COT sequence does poorly in terms of total coverage (unpublished data). This observation is due to the fact that many of the 9mers do not overlap, and therefore the fragments cannot be efficiently joined. By going back and selecting high-coverage peptide windows from the original data, we obtain better compression in the vaccine construct leading to higher coverage constructs for the same length.

**Figure 5:**
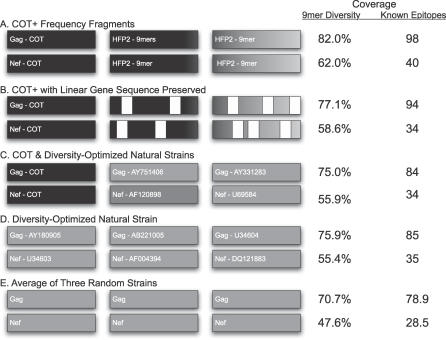
Possible Configurations for Vaccine Constructs Each bar represents one unit-length gene. The fill intensity of each bar represents the density of unique peptides and known CTL epitopes. The coverage that each construct captures of the amino acid diversity of the dataset is shown on the right for both 9mers and epitopes. (A) COT^+^, composed of the estimated COT plus the appended high-frequency peptides (HFPs) composing the second and third gene lengths. (B) COT plus HFPs placed into a gene collinear fashion on the second and third gene lengths. (C) COT plus two NSs chosen to maximize 9mer coverage. (D) All NSs of Gag and Nef sequences chosen such that 9mer coverage is maximized and for comparative reasons (E) is average coverage across all NSs. The GenBank IDs of the NSs are written inside each bar.

It is a reasonable assumption that the retention of native protein structures might be advantageous in generating CTL epitopes, since epitopic peptides are generated in vivo by protein degradation within infected cells. Nef and Gag COT clearly adopt a native structure, as they retain biological activity (Rolland M, Jensen MA, Nickle DC, Learn GH, Heath L, et al., unpublished data). However, the extended COT^+^ component of antigens generated in the manner proposed here does not preserve a sequence that is necessarily collinear with the native gene over the second and third gene lengths (Figure 5A). Hence, we have also considered additional means of optimizing immunogen structures that also preserve native structure. First, we can assemble high-frequency variable elements in a pattern collinear with the native gene, with some segments redundant with COT to retain collinearity (Figure 5B). We can also use NS sequences in combination with the COT sequence to optimize coverage (Figure 5C). We can also do very well in generating coverage by exclusive use of NS sequences that maximize 9mer coverage (Figure 5D). Although it is not guaranteed, these additional constructs (Figure 5B–5D) should have biologically acceptable tertiary structures. The COT^+^ approach captures more of the 9mer distribution and more of the known CTL epitopes than any of the potential constructs presented here. Applying high-frequency peptides onto COT to create a collinear pattern provides the second highest level of diversity and epitope enrichment, but the use of COT plus two NSs is not beneficial relative to judicious choice of three NSs. Last, it should be noted that all of these methods substantially exceed the coverage afforded by the use of a single strain as a vaccine.

Immunodominance gives rise to a rank order of immune responses to specific epitopes [28], and the underlying biological mechanisms giving rise to these rank orders are poorly understood. The antigen designs we report here do not take immunodominance into account. One can argue that the combination of epitopes we have derived could elicit an immunodominant response that does not reflect what is found in circulating HIV strains and hence could be a poor choice for vaccine design. However, since the strings of peptides in our immunogen design are captured by their frequency in the circulating viral population, we surmise that these antigens have epitopes that are shared across many potential challenge strains and could thus lead to potentially broad immune response. However, immunodominance rank order patterns can be partially illuminated by expressing epitopes from different vaccine vectors [29–31]. By vaccinating with different combinations of vectors encoding a single or more antigens, they found that using separate vectors elicited broader CD8^+^ T cell responses. Because COT^+^ is directed towards capturing high-frequency fragments from a variable protein, it is well-suited to being expressed as segments on separate vectors. The COT^+^ algorithm can be generalized to produce *sets* of immunogens that can take advantage of this phenomenon.

COT^+^ constructs are able to capture significantly more known epitopes and potential antigen variability than much longer constructs composed by combining circulating strains. Considering the substantial expense and difficulty involved in production and testing of candidate vaccines, careful crafting of potential antigens using computational methods, including that shown here, may be beneficial. Furthermore, this approach is applicable not only to HIV vaccine design, but to the design of vaccines targeting any pathogen capable of rapid escape from immune recognition.

## Abbreviations

ANC: ancestors
CON: consensus
COT: center of tree
CTL: cytotoxic T lymphocyte
HIV-1: human immunodeficiency virus type 1
NS: natural strain
